# Comparative proteomic analysis of early salt stress-responsive proteins in roots of SnRK2 transgenic rice

**DOI:** 10.1186/1477-5956-10-25

**Published:** 2012-03-31

**Authors:** Myung Hee Nam, Sun Mi Huh, Kyung Mi Kim, Woong June Park, Jong Bok Seo, Kun Cho, Dool Yi Kim, Beom Gi Kim, In Sun Yoon

**Affiliations:** 1 Bio-Crops Development Division, National Academy of Agricultural Sciences, Suwon 441-857, Republic of Korea; 2 Seoul Center, Korea Basic Science Institute, Seoul 136-701, Republic of Korea; 3 Department of Molecular Biology & Institute of Nanosensor and Biotechnology, Dankook University, Yongin-si, Gyeonggi-do 448-701, Republic of Korea; 4 Division of Mass Spectrometry, Korea Basic Science Institute, Ochang 363-883, Republic of Korea

## Abstract

**Background:**

The rice roots are highly salt-sensitive organ and primary root growth is rapidly suppressed by salt stress. Sucrose nonfermenting 1-related protein kinase2 (SnRK2) family is one of the key regulator of hyper-osmotic stress signalling in various plant cells. To understand early salt response of rice roots and identify SnRK2 signaling components, proteome changes of transgenic rice roots over-expressing OSRK1, a rice SnRK2 kinase were investigated.

**Results:**

Proteomes were analyzed by two-dimensional electrophoresis and protein spots were identified by LC-MS/MS from wild type and OSRK1 transgenic rice roots exposed to 150 mM NaCl for either 3 h or 7 h. Fifty two early salt -responsive protein spots were identified from wild type rice roots. The major up-regulated proteins were enzymes related to energy regulation, amino acid metabolism, methylglyoxal detoxification, redox regulation and protein turnover. It is noted that enzymes known to be involved in GA-induced root growth such as fructose bisphosphate aldolase and methylmalonate semialdehyde dehydrogenase were clearly down-regulated. In contrast to wild type rice roots, only a few proteins were changed by salt stress in OSRK1 transgenic rice roots. A comparative quantitative analysis of the proteome level indicated that forty three early salt-responsive proteins were magnified in transgenic rice roots at unstressed condition. These proteins contain single or multiple potential SnRK2 recognition motives. In vitro kinase assay revealed that one of the identified proteome, calreticulin is a good substrate of OSRK1.

**Conclusions:**

Our present data implicate that rice roots rapidly changed broad spectrum of energy metabolism upon challenging salt stress, and suppression of GA signaling by salt stress may be responsible for the rapid arrest of root growth and development. The broad spectrum of functional categories of proteins affected by over-expression of OSRK1 indicates that OSRK1 is an upstream regulator of stress signaling in rice roots. Enzymes involved in glycolysis, branched amino acid catabolism, dnaK-type molecular chaperone, calcium binding protein, Sal T and glyoxalase are potential targets of OSRK1 in rice roots under salt stress that need to be further investigated.

## Background

Salinity is a major constraint to crop productivity. Plant salt tolerance involves diverse mechanisms such as osmolyte accumulation, ion homeostasis, cellular protection from damage by reactive oxygen species, growth regulation, and signal perception and transduction [1-4]. As plant roots are primary site of perception and highly sensitive organ to salt stress, the understanding for roots to salt response can contribute to increasing the crop productivity. So far, numerous salt-regulated genes and proteins were identified by microarray and proteomic studies in roots of various plants such as tomato, *Arabidopsis*, tobacco and rice [5-9]. The identified salt-responsive proteins are involved in diverse cellular functions such as regulation of carbohydrate, nitrogen and energy metabolism, ROS scavenging, detoxification, signal transduction, RNA and protein processing and cytoskeleton. On the other hands, large numbers of salt responsive genes reported in the literature have not been identified so far by proteomic approaches, and mRNA level was not correlated well with the protein level. Therefore, characterization of post-transcriptional and post-translational regulatory systems is crucial for the deeper understanding of the molecular mechanisms governing plant adaptation to salt stress. Phosphorylation is one of the best known post-translational protein modifications affecting conformation, activity, localization and stability of target proteins [10]. Under salt stress, protein phosphorylation cascades are activated and play a critical role in rice salt tolerance [11-15]. However, phosphoproteome or proteome changes related to specific protein kinase signaling under salt stress have not been well characterized.

Protein kinases function as key regulators of salt stress and ABA signaling in plants. Diverse protein kinase families such as mitogen-activated protein kinases (MAPK), calcium-dependent protein kinases (CDPK), SNF1-related protein kinases (SnRK) and receptor like kinases (RLKs) were found to be activated by ABA and diverse stress signals [13,16-20]. Arabidopsis SOS2 (salt overly sensitive2), the calcium sensor-associated SnRK3 family, regulates sodium ion homeostasis and is required for salt tolerance [21]. This SOS salt tolerant pathway is likely to be conserved in cereals such as rice [14]. Transgenic plants over-expressing rice OsCIPK3, OsCIPK12, and OsCIPK15 showed significantly improved stress tolerance to cold, drought, and salt stress, respectively [22].

Osmotic stress or ABA treatment rapidly activates 42-48 kDa protein kinases, known as SnRK2 family later [17,19,23-26]. Biochemical studies indicated that all rice and Arabidopsis SnRK2 kinases except AtSnRK2.9 are activated by salt treatment [15,27], implicating that they may have important function in plant salt responses. On the other hand, several SnRK2 members of Arabidopsis (2.2, 2.3, 2.6, 2.7 and 2.8) and rice (SAPK8, SAPK9 and SAPK10) were activated by ABA as well [15,27]. It has been appeared that these ABA-activated SnRK2 subfamily members are key regulators of ABA signaling pathway function in control of seed development, dormancy, seed germination, seedling growth as well as stomata regulation in drought response [28-30]. Recent progress showed that Arabidopsis SnRK2.6/OST1 is a core component of ABA signal transduction pathway leading to activation of an ion channel SLAC1 in guard cells [31] and ABF-induced gene expression in plant cell [32]. In spite of the recent progress in those limited number of SnRK2 signaling pathway in ABA responses of Arabidopsis, little is known about the function of other SnRK2 kinase members, in particularly, associated with salt stress signaling. There is a report showing that over-expression of a rice SnRK2 kinase SAPK4 regulates salt stress response [33].

OSRK1/SAPK6 is a rice SnRK2 kinase function in ABA and hyperosmotic stress signaling [15,34]. In this study, proteome analysis was conducted in the transgenic rice over-expressing OSRK1 to identify OSRK1-regulated proteome changes associated with early salt stress responses of rice roots. Two-DE based comparable proteomic analysis and LC-MS/MS were applied to identify protein changes during the early response of roots to salt stress. Proteomic analysis indicates that OSRK1 is an upstream regulator of the salt stress responses associated with regulation of carbon, nitrogen and energy metabolism, and detoxifying enzymes in rice roots.

## Results and discussion

### Phosphorylation activity in roots of OSRK1 transgenic rice

**Figure 1:**
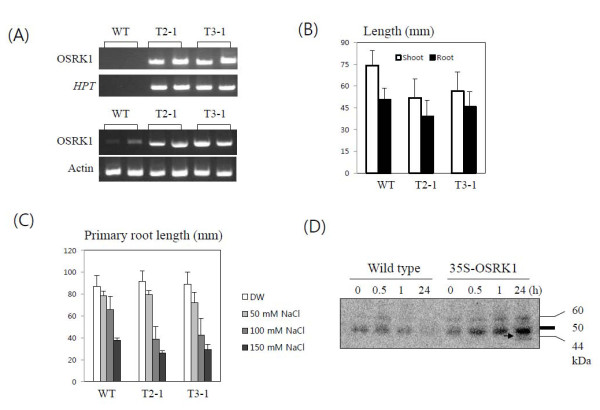
**Phenotypes of OSRK1 transgenic rice**. (A) PCR analysis of OSRK1 transgenic rice. Insertion and expression of the transgene was confirmed by genomic PCR (upper) and RT-PCR analysis (bottom). The PCR primers for OSRK1 were designed to specifically detect transgene or mRNA. RT-PCR analysis indicated that transcript level of OSRK1 was greatly increased in the transgenic rice roots. (B) Comparison of shoot and root growth of OSRK1 transgenic rice. Root and shoot length was measured after 9 days-growth on 1/2 MS agar media. (C) Effect of NaCl on the primary root growth of wild type and transgenic rice roots. Germinating seedlings with equally growing primary roots were selected and primary root length was measured 5 days after NaCl treatment at indicated concentrations. (D) In-gel kinase assay. Total soluble proteins were extracted from the roots of wild type or OSRK1 transgenic rice seedlings after 150 mM NaCl treatment for the indicated time period. Histone was used as a substrate.

OSRK1/SAPK6 showed strong kinase activity *in vitro *and rapidly activated by salt and osmotic stress when transiently expressed in rice suspension cells [15,34]. In an attempt to identify OSRK1 signaling pathway, we have generated transgenic rice over-expressing OSRK1 under the control of CaMV 35S promoter. Insertion and expression of the transgene was confirmed by genomic PCR and Northern blot analysis (Figure 1A). Compared to wild type, seedling growth of the transgenic rice was retarded (Figure 1B), and plant height was significantly decreased under paddy field growth condition (data not shown). We often observed root growth of the transgenic rice was impaired depending on the growth condition (data not shown). It was found that primary root elongation of OSRK1 transgenic rice was more sensitive to NaCl stress (Figure 1C).

Phosphorylation activity in wild type and transgenic rice roots in response to NaCl was compared. In gel kinase assay with histone as a substrate detected two phosphorylation bands approximately at 52 kDa and 61 kDa, and these kinase activities were transiently increased by NaCl treatment in wild type roots (Figure 1D). In transgenic rice roots, the signal intensity of these bands was much stronger and remained for longer time (Figure 1D). Our data indicates that protein phosphorylation activity was significantly increased by over-expression of OSRK1 in rice roots. Whether the 52-60 kDa salt-responsive kinases detected in our in gel kinase assay are targets of OSRK1 remains to be elucidated.

### Proteome analysis of early salt-responsive proteins in OSRK1 transgenic rice roots

**Figure 2:**
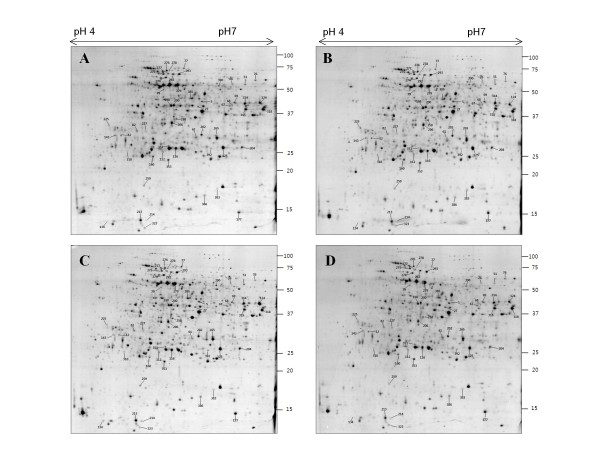
**Protein expression profiles in response to a salt treatment in OSRK1 transgenic rice roots**. Proteins were extracted from transgenic rice roots after treatment with 150 mM NaCl (B, D) or control medium (A, C) for 3 h (A, B) and 7 h (C, D). Spot numbers indicated the identified protein spots describe in Table 1, that their abundance was increased or decreased following NaCl treatment at 3 h or 7 h.

**Figure 3:**
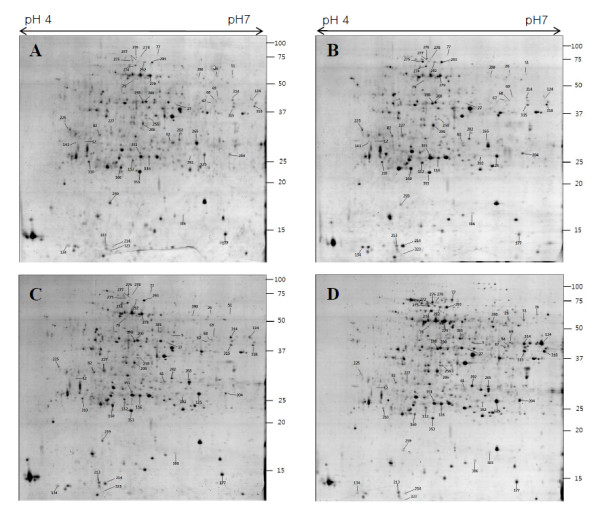
**Protein expression profiles in response to a salt treatment in WT rice roots**. Proteins were extracted from WT rice roots after treatment with 150 mM NaCl (B, D) or control medium (A, C) for 3 h (A, B) and 7 h (C, D). Spot numbers indicated the identified protein spots described in Table 1, that their abundance was increased or decreased following NaCl treatment at 3 h or 7 h.

Rice roots are very sensitive organ to sense salt stress, and could be a suitable system to investigate OSRK1-dependent targets related to salt stress response. As we found that over-expression of OSRK1 resulted in high phosphorylation activity and salt-sensitive root growth (Figure 1C and 1D), proteomic analysis was carried out to monitor the salt response of OSRK1 transgenic rice roots at protein level. Protein kinase activities of many plant SnRK2 family members were known to be rapidly activated by salt stress [15,17,19,23-27]. We therefore focussed on the initial changes of protein profiles in order to identify OSRK1-mediated salt stress signalling components. Two-DE based comparative proteomic analysis was performed in wild type and OSRK1 transgenic rice roots with 3 h or 7 h of salt treatment (Figure 2 and 3).

Previous reports have presented diverse proteome profiles changed by salt stress in plant roots [5,8,9,35-37]. Contrary to our expectation, however, only seven salt-responsive protein spots were detected as an significant different (P = 0.05) from control group in the transgenic rice roots either by 3 h or 7 h salt stress (Figure 2 and Table 1). They are two translation elongation factors (spot 5 and 12), dihydrolipoamide dehydrogenase (spot 76), glutathione S-transferase, proteasome subunit beta type 2 and/or chaperonin21 precursor (spot 112), ascorbate peroxidase (spot 160), 15 kda Organ-specific salt-induced protein and/or inorganic pyrophosphatase (spot 227), and glyoxalase II (spot 265). With less significant difference (P = 0.05 ~ 0.07), dnaK-type molecular chaperone precursor (spot 276, 278) and enolase (spot 279) is changed by salt treatment (Figure 2 and Table 1). Most of these proteins were salt-responsive in wild type rice roots as well (Table 1), and proteins specific to the transgenic rice were not found.

**Table 1:** Identification and quantitative analysis of early salt-responsive proteins in wild type and OSRK1 transgenic rice roots

Spot^a^	Protein Identification	Matched peptide	Sequence Coverage (%)	M*r*^b^ (Ex/Tr)	NCBI Accession Number	Fold change^c^				Protein ratio^d^ (OSRK1/WT)	Express Graph^e^
								
						WT		OSRK1			
								
						3 h	7 h	3 h	7 h		
**Translation and transcription**											
5^i^	Elongation factor Tu	9	26.3	43.7/48.4	gi|21685576	nd^f^	7.32	2.47	1.19	I^g^	
12^i^	Putative translation elongation factor eEF-1 beta chain	5	35.3	30.0/23.8	gi|113612079	0.97	2.00	1.92	0.55	1.25	
323	Glycine-rich RNA-binding protein	6	74.0	13.1/15.5	gi|115441831	1.02	0.38	1.14	0.85	0.93	
383	Translation initiation factor 5A	2	25.0	17.7/17.4	gi|113611710	nd	2.84	1.04	0.62	I^g^	
**Amino acid and purine metabolism**											
26	Putative inosine monophosphate dehydrogenase	2	4.70	57.4/51.9	gi|215694434	0.38	1.25	0.89	0.88	1.05	
27	Aspartate-semialdehyde dehydrogenase	3	10.9	40.1/40.1	gi|113549818	2.11	3.05	1.15	0.80	5.46	
67	Aspartate aminotransferase	4	13.0	40.6/44.8	gi|29468084	1.48	4.51	0.87	0.97	3.64	
68	Putative isovaleryl-CoA dehydrogenase	1	2.70	41.5/44.5	gi|113578072	0.88	1.89	0.91	0.89	2.53	
200	Glutamine synthetase	4	10.4	41.4/39.2	gi|124052115	1.51	1.37	0.98	1.02	1.72	
259	Methylmalonate semi-aldehyde dehydrogenase	5	10.5	18.1/57.2	gi|113610618	0.46	0.60	1.22	0.67	0.27	
314	Aspartate aminotransferase	6	18.8	42.2/44.9	gi|215768565	1.13	3.14	0.87	1.06	3.08	
314	Glutamate dehydrogenase	7	21.4	42.2/44.3	gi|33242905	1.13	3.14	0.87	1.06	3.08	
**Detoxyfying enzymes**											
51	Glutathione reductase, cytosolic	11	24.8	55.6/53.4	gi|113538016	0.63	2.80	0.89	1.01	1.82	
112^i^	Gutathione S-transferase	4		25.6/25.6	gi|31433227	1.67	0.72	1.35	1.51	0.28	
116	Ascorbate peroxidase	5	33.6	25.8/27.1	gi|50920595	1.79	1.13	0.80	1.10	2.40	
125	Glutathione S-transferase II	6	32.1	26.0/24.0	gi|3746581	2.05	1.42	0.94	0.90	2.63	
160^i^	Ascorbate peroxidase	10	46.0	24.0/27.1	gi|1321661	1.35	0.38	1.51	0.71	0.41	
198	Quercetin 3-O-methyltransferase	2	6.8	41.4/39.7	gi|113623000	1.57	1.19	1.06	0.84	1.83	
200	Peroxidase	3	8.6	41.4/37.8	gi|257657027	1.51	1.37	0.98	1.02	1.72	
202	Similar to glyoxalase II	6	28.2	29.9/33.1	gi|113533338	1.07	1.98	0.94	0.90	1.47	
206	Glyoxalase I	10	51.5	33.9/32.5	gi|113623141	3.57	1.73	0.84	1.00	4.46	
213	Thioredoxin Type H	7	50.8	13.6/13.9	gi|82407383	1.94	0.87	1.01	0.98	3.20	
265^i^	Similar to glyoxalase II	7	32.5	32.5/33.2	gi|113533338	0.59	1.52	0.68	0.74	1.90	
310	Ascorbate peroxidase	5	38.2	25.6/27.1	gi|257707656	0.79	0.34	1.85	4.40	0.10	
351	Ascorbate peroxidase	4	24.8	26.7/27.1	gi|1321661	1.98	0.65	1.00	0.97	1.35	
390	Aldehyde dehydrogenase	3	8.0	54.6/59.2	gi|8163730	3.16	3.10	0.85	1.25	10.85	
**Glycolysis and other carbohydrate metabolism related proteins**											
61	Fructose -bisphosphate aldolase	3	9.8	29.3/38.8	gi|790970	0.26	0.94	0.69	0.81	0.82	
67	Phosphoglycerate kinase	3	11.0	40.6/42.3	gi|113596357	1.48	4.51	0.87	0.97	3.64	
76^i^	Dihydrolipoamide dehydrogenase precursor	5	13.5	60.6/52.6	gi|113532449	nd	16.87	0.87	1.54	I^g^	
77	Transketolase	7	10.6	76.8/80.0	gi|227468492	2.14	15.09	1.02	0.98	18.68	
79	Similar to enolase	6	15.9	52.0/50.7	gi|115478881	1.90	0.78	0.80	0.74	3.14	
116	Triose phosphate isomerase	13	79.1	25.8/27.6	gi|553107	1.79	1.13	0.80	1.10	2.40	
275	2,3-Bisphosphoglycerate-independent phosphoglycerate mutase	8	27.2	70.8/60.8	gi|257353836	1.45	2.56	0.80	0.84	2.07	
277	2,3-Bisphosphoglycerate-independent phosphoglycerate mutase	3	12.2	70.8/60.8	gi|257353836	1.26	2.83	0.86	0.88	2.33	
279^i^	Enolase	4	14.4	57.0/47.9	gi|113548027	1. 03	1.70	0.72	0.80	1.41	
292	Phosphoglyceromutase	9	20.8	69.9/60.7	gi|257353838	1.03	2.13	0.93	1.04	2.66	
293	Phosphoglyceromutase	12	28.1	69.9/60.7	gi|257353838	1.50	1.79	0.87	1.02	1.90	
315	Glyceralde-3-phosphate dehydrogenase	4	16.3	39.1/36.5	gi|968996	0.99	2.14	0.91	1.10	2.15	
318	Glyceralde-3-phosphate dehydrogenase	4	16.3	38.7/36.4	gi|968996	1.17	2.68	1.03	1.01	3.06	
353	Triose phosphate isomerise	4	20.9	22.0/27.0	gi|169821	0.83	0.62	1.16	0.67	0.32	
385	Phosphoglycerate kinase	5	16.7	45.4/42.2	gi|113596357	nd	4.57	1.03	1.10	I^g^	
**Proteolytic enzymes**											
112^i^	Proteasome subunit beta type 2	5	26.4	25.6/23.4	gi|17380213	1.67	0.72	1.35	1.51	0.28	
134	Oryzacystatin	3	23.5	13.3/11.4	gi|1280613	2.10	0.46	0.87	1.00	0.22	
204	Proteasome subunit alpha type 2	7	34.6	26.2/27.6	gi|259443357	0.59	1.88	0.89	1.15	1.22	
351	Proteasome subunit alpha type 2	4	16.6	26.7/25.8	gi|259443327	1.98	0.65	1.00	0.97	1.35	
**Heat shock proteins**											
112^i^	Putative chaperonin21 precursor	5	26.2	25.6/26.3	gi|51090748	1.67	0.72	1.35	1.51	0.28	
274	Chaperonin CPN60-1, mitochondrial precursor	10	17.5	64.1/61.0	gi|113547409	1.17	2.77	0.88	1.12	1.49	
276^i^	DnaK-type molecular chaperone precursor	2	4.6	75.0/70.4	gi|257307253	1.26	3.35	0.76	1.00	2.78	
278^i^	DnaK-type molecular chaperone precursor	6	14.5	72.9/70.4	gi|257307253	2.35	2.38	0.66	1.47	4.47	
**Lipid biosynthesis**											
258	Putative enoyl-ACP reductase	3	15.7	35.4/39.1	gi|113623526	1.32	2.41	1.13	1.01	2.92	
69	Putative acetyl-CoA C-acetyltransferase	6	19.2	43.1/44.1	gi|113630918	1.28	3.46	0.80	0.99	6.40	
82	Putative inorganic pyrophosphatase	5	21.4	31.8/33.0	gi|113537770	0.88	0.41	0.97	0.99	0.42	
177	Nucleoside diphosphate kinase from rice	2	16.6	14.5/16.8	gi|113639936	0.39	0.81	0.88	0.83	1.14	
227^i^	Putative inorganic pyrophosphatase	5	13.9	32.1/33.0	gi|113537770	1.70	0.41	0.92	1.30	0.44	
**Stress related proteins**											
124	Formate dehydrogenase, mitochondrial precursor	13	47.2	42.4/41.2	gi|21263611	0.64	7.24	0.78	0.90	4.13	
134	15 kda Organ-specific salt-induced protein	2	23.4	13.3/15.2	gi|256638	2.10	0.46	0.87	1.00	0.22	
214	15 kda Organ-specific salt-induced protein	7	77.2	13.4/15.2	gi|256638	4.05	0.33	0.87	1.12	0.48	
227^i^	15 kda Organ-specific salt-induced protein	6	64.8	32.1/15.2	gi|256638	1.70	0.41	0.92	1.30	0.44	
386	Pathogen-related protein	4	21.3	16.5/17.2	gi|16589076	4.29	2.28	1.30	0.38	6.70	
**Signal transduction related proteins**											
225	Calreticulin	3	5.9	33.4/47.9	gi|6682833	0.75	0.43	1.47	1.26	0.26	
**Unkwown proteins**											
392	Hypothetical protein	3	10.7	25.8/27.4	gi|14192878	5.65	4.00	0.79	0.96	18.95	

^a^Spot numbers correspond to the spots in Figure 2 and Figure 3

^b^Molecular weight (kDa) of protein spots estimated from gel analysis (Ex) and theoretical molecular weight of identified protein (Tr)

^c^Fold change of each spot after NaCl treatment for 3 h or 7 h, calculated from the mean spot volumes in control and NaCl treated gel groups

^d^Spot-volume ratio of WT to OSRK1, calculated from the mean spot volumes in 3 h control of WT and OSRK1 groups

^e^Relative expression graphs of protein spots after NaCl treatment in WT and OSRK1. Spot volumes are analyzed by Progenesis software. The left four bars indicate spot volume of WT root and right four bars indicate spot volumes of OSRK1. From left to right, each bar indicate spot volume of 3 h control, 3 h NaCl treated, 7 h control and 7 h NaCl treated gel groups. Values are means ± SE. Error bars from three spots in three independent gels

^f^nd: not detected

^g ^I: spots were not detected in WT groups, but appeared in OSRK1 groups

^h^Proteins not identified from the database

i Salt-responsive proteome identified from OSRK1 transgenic rice roots

### Proteome analysis of early salt-responsive proteins in wild type rice roots

Because the salt-responsive proteome changes in transgenic rice were unexpectedly low, proteome changes in wild type and transgenic rice roots were compared. Figure 3 shows the 2-DE gel images of proteins extracted from control and NaCl-treated WT roots. We identified 52 spots which were changed their abundance (vol %) more than 1.5 fold by either 3 h or 7 h salt treatment (Table 1). Of the 52 spots identified, 8 spots (15.4% of identified proteins) were found to have more than two unrelated proteins, indicating the presence of multiple proteins in one spot. Some proteins were found in multiple spots. These proteins were aspartate aminotransferase (spot 67 and 314), inorganic pyrophosphatase (spot 82 and 227), ascorbate peroxidase (spot 310 and 353), 15 kDa organ-specific salt-induced protein (spot 134, 214 and 227), glyoxalase II (spot 202 and 265), proteasome subunit alpha (spot 204 and 351), 2,3-bisphosphoglycerate-independent phosphoglycerate mutase (spot 275 and 277), dnaK-type molecular chaperone (spot 276 and 278), phosphoglyceromutase (spot 292 and 293), glyceralde-3-phosphate dehydrogenase (spot 315 and 318), phosphoglycerate kinase (spot 67 and 385) and triose phosphate isomerase (spot 116 and 353). These multiple spots on 2-DE gel are presumably due to post-transcriptional modification, or expression of differential isoforms derived from different genes, or proteolytic degradation of proteins *in vivo *and *in vitro*. Of the 52 salt-responsive protein spots identified, 6 spots were down-regulated and 16 were up-regulated more than 1.2 fold by 3 h and 7 h of salt treatment. Others are transiently up or down-regulated at 3 h or changed only after 7 h. The identified early salt-responsive proteins were classified into 11 functional categories (Table 1). Similar to the previous reports on the salt-responsive proteins of Arabidopsis and rice [5,8,9,36,37], enzymes related to energy metabolism, primary metabolism and detoxification were major protein families rapidly changed by salt stress. In addition, proteins related to translation, proteolytic enzymes and heat shock proteins were significantly changed by salt stress. Other proteins related to lipid biosynthesis, RNA process, stress-related and signal transduction were identified as well.

Our current proteome data indicated that rice roots rapidly changed broad spectrum of energy metabolism upon challenging salt stress. The major up-regulated proteomes were metabolic enzymes related to glycolysis, pentose phosphate pathway, ammonium assimilation, aspartate pathway, branched amino acid breakdown, lipid synthesis, methylglyoxal detoxification and redox regulation. Change in the anerobic respiratory metabolism is a key component of early salt response of rice roots and glycolysis may play central role. It is noteworthy that several GA-responsive proteomes [38-40] were clearly down-regulated in rice roots by salt stress. They are fructose-bisphosphate aldolase (spot 61), inorganic pyrophosphatase (spot 82 and 227), oryzasystatin (spot 134), 15 kDa organ-specific salt induced protein (spot 134, 214 and 227), calreticulin (spot 225) and methylmalonate semi-aldehyde dehydrogenase (MMADH, spot 259). Among these, fructose bisphosphate aldolase and calreticulin were also identified as proteins down-regulated by ABA, a GA antagonistic hormone [41-43]. GA is a key regulator of root cell elongation and development of rice. It was reported that aldolase and MMADH function in GA-induced root growth in rice [38,40]. Suppression of GA signaling by salt stress may responsible for arrest of rice root growth and development under salinity condition.

#### Glycolysis and carbohydrate metabolism

Salt stress is known to induce a decrease in the oxygen uptake in several plants [44]. At the oxygen limiting circumstances, glycolytic pathway is activated to maintain cellular homeostasis and energy production [45]. Coordinate regulation of the expression of different glycolytic enzymes contribute to maintaining homeostasis in rice cells under oxygen deprivation [46]. Glycolytic gene expression was increased in rice shoots and roots under various abiotic stresses condition including salt stress [45]. Previous rice proteome analysis revealed that glycolytic enzymes were up-regulated by long-term salt stress [8,36]. Our proteome analysis further confirmed that glycolytic pathway is rapidly activated at early phase of salt response of rice roots. Five glycolytic enzymes such as triosephosphate isomerase (spot 116), glyceralde-3-phosphate dehydrogenase (spot 315 and 318), phosphoglycerate kinase (spot 67 and 385), 2,3-bisphosphoglycerate-independent phosphoglycerate mutase (spot 275 and 277), and enolase (spot 79 and 279) were increased at either 3 h or 7 h salt treatment (Table 2). In addition to these enzymes, dramatic increase of transketolase (spot 77) and formate dehydrogenase (spot 124) was observed (Table 2). Transketolase is known as the key enzyme regulating flux between pentose phosphate pathway and glycolysis in E. coli. In plants, transketolase is an essential enzyme of the Calvin cycle. When the transketolase was repressed in tobacco, dramatic changes in photosynthesis and phenylpropanoid metabolism were induced, indicating the central role of transketolases in the primary metabolism [47]. Formate dehydrogenase is an enzyme of anaerobic metabolism and the protein was increased in stressed potato [48,49]. Glycolysis may play an important role in formate synthesis [48]. Taken together, these data indicate that anaerobic metabolism is a key component of early salt response of rice roots and glycolysis play an important role. The cytosolic network of glycolytic enzymes may provide an essential metabolic flexibility that facilitates plant developments and acclimation to environmental stress [49]. Glycolysis also has a role in the production of a wide range of metabolites including amino acids, lipids and related compounds [45,49].

**Table 2:** The potential SnRK2 phosphorylation sites found in protein spots showing more than 1.5 fold change in OSRK1 transgenic rice roots at unstressed condition

Spot^a^	Protein Identification	NCBI Accession #	Potential recognition motifs (SnRK2)^b^	S^c^	T/W^d^
5	Elongation factor Tu	gi|21685576	RRILS, RGIT	U	I
27	Aspartate-semialdehyde dehydrogenase	gi|113549818	**RRPSS**, **FLRVIS**	U	U
61	Fructose-bisphosphate aldolase	gi|790970	RFAS	D	D
67	Aspartate aminotransferase	gi|29468084	RVAT, RVKS	U	U
68	Putative isovaleryl-CoA dehydrogenase	gi|113578072	RRLYS, **LVRHGS**	U	U
69	Putative acetyl-CoA C-acetyltransferase	gi|113630918	RSSS, RKGS	U	U
76	Dihydrolipoamide dehydrogenase precursor	gi|113532449	RLGS, RFMT	U	I
77	Transketolase	gi|227468492	RNLS, RVVS	U	U
79	Simillar to enolase	gi|115478881	RAAT, RNQS, RMGS	U	U
82, 227	Putative inorganic pyrophosphatase	gi|113537770	**RRRCSLRTNS**, RKVS	D	D
112	Glutathione S-transferase	gi|31433227	Not found	U	D
112	Proteasome subunit beta type 2	gi|17380213	RRAYT	U	D
112	Putative chaperonin21 precursor	gi|51090748	RVCS, RRPS	U	D
116	Triose phosphate isomerise	gi|553107	Not found	U	U
124	Formate dehydrogenase, mitochondrial precursor	gi|21263611	Not found	U	U
125	Glutathione S-transferase II	gi|3746581	**MARPSS**	U	U
134	Oryzacystatin	gi|1280613	Not found	D	D
134, 214, 227	15 kda organ-specific salt-induced protein	gi|256638	**FGRSGT**	D	D
160	Ascorbate peroxidise	gi|1321661	Not found	D	D
200	Peroxidase	gi|257657027	**IDRAKS**, RNDS	U	U
200	Glutamine synthetase	gi|124052115	RTLS, RRLT, RHET, RGAS, RPAS	U	U
202, 265	Putative glyoxalase II	gi|113533338	**RARPIS**	U	U
206	Glyoxalase I	gi|113623141	**IQRGPT**	U	U
213	Thioredoxin type H	gi|82407383	Not found	U	U
225	Calreticulin	gi|6682833	**RARSSS**	D	D
258	Putative enoyl-ACP reductase	gi|113623526	**IGRALS**, RAMS, RVNT	U	U
259	Methylmalonate semi-aldehyde dehydrogenase	gi|113610618	**LLRSGS**, RVQS, RDAT, **VKRASS**	D	D
275,277	2,3-bisphosphoglycerate-independent phosphoglycerate mutase	gi|257353836	RSET, **VKRNKS**	U	U
276, 278	DnaK-type molecular chaperone precursor	gi|257307253	**FARTFS**, RQAT, **INRNTT**	U	U
292, 293	Phosphoglyceromutase	gi|257353838	RIAS, RAET, **VKRNKS**	U	U
314	Glutamate dehydrogenase	gi|33242905	**LTRVFT**	U	U
314	Aspartate aminotransferase	gi|215768565	RLPT	U	U
315, 318	Glyceralde-3-phosphate dehydrogenase	gi|968996	RAAS, RVPT	U	U
385, 67	Phosphoglycerate_kinase	gi|113596357	Not found	U	U
383	Translation initiation factor 5A	gi|113611710	RLPT	U	I
386	Pathogen-related protein	gi|16589076	Not found	U	U
390	Aldehyde dehydrogenase	gi|8163730	**RRGSS**, **LQRFST**, RVGT	U	U
392	Hypothetical protein	gi|14192878	**VLRLRS**, RYAT	U	U

^a^Spot numbers correspond to the spots in Figure 2

^b^Potential phosphorylation motifs for SnRK2 kinase, RXX(S/T) were analyzed. Motifs with favouring amino acids (L, I, V, M, F, R) at -5 position for CDPK/SnRK2 were marked as bold characters according to Vlad et al. (2008). Among these, the highly preferred motifs, (L/I)XRXX(S/T) were underlined

^c^Up (U) or down (D)-regulated protein spots in wild type rice roots after NaCl treatment for 3 h or 7 h

^d^Up (U) or down (D)-regulated or induced (I) protein spots in OSRK1 transgenic rice roots compared to wild type roots at 3 h or 7 h control

#### ROS regulation and detoxifying enzymes

Reactive oxygen species (ROS) are regarded as the main source of cell damage under abiotic stresses including salt stress [50]. Plants have developed protective mechanisms to eliminate or reduce ROS, and the enzymatic antioxidant system is one of the protective mechanisms [51]. We identified several ROS removing and redox regulating proteins up-regulated by salt treatment in rice roots (Table 1). The rapid increase of an ascorbate peroxidase (APX, spot 116 and 351) and glutathione reductase (GR, spot 51) by salt stress is consistent with the previous report that the activity and transcript levels of these proteins were increased within 4 h or 0.5 h in salt treated rice roots [51-53]. Increase in activity of antioxidant enzymes, such as ascorbate peroxidase, catalase and glutathione reductase, can contribute to salt tolerance of potato [54]. On the other hand, another APX spot (spot 310) was found to be decreased by salt stress (Table 1). This differential response of APXs by salt stress was observed in Arabidopsis roots and may reflect sub-functionalization of APX gene families at the regulatory and catalytic levels [55].

Glyoxalase I (GlyI, spot 206) and glyoxalase II (Gly2, spot 202 and 265) were increased by salt stress. These two enzymes are related to glyoxalase pathway which is required for glutathione-based detoxification of methylglyoxal (MG). The glyoxlase transcript level is rapidly induced by salt stress and closely correlated to salt tolerance of tomato [56,57]. Over-expression of glyoxalase pathway genes in transgenic plants has been found to keep a check on the MG level under stress conditions, regulate glutathione homeostasis, and the transgenic plants are able to survive and grow under various abiotic stresses [58]. In rice roots, glyoxalase I was identified as a salt-induced proteome [36]. Recent report showed that MG detoxification system (enzyme activity of glyoxalase I and II) was significantly higher in salt tolerant Pokkali rice [59]. Transgenic rice over-expression of glyoxalse II showed enhanced tolerance to toxic concentration of MG and NaCl, implicating functional importance of this enzyme in salt tolerance of rice [58].

Thioredoxin H-type protein (spot 213) and aldehyde dehydrogenase (spot 390) were identified as salt up-regulated proteins. Thioredoxin H is a protein involved in redox regulation by reducing disulfide bridges [60]. It has been known that thioredoxin H is induced under oxidative stress condition [61]. Recently, thioredoxin H-type protein was identified as a salt-responsive apoplastic protein in rice roots [62]. Aldehydes are intermediates in several fundamental metabolism pathways for carbohydrates, vitamins, steroids, amino acids, and lipids [63]. They are also phytotoxic metabolites produced by stresses that disturb metabolism, including salinity [64,65]. By catalyzing the irreversible oxidation of a wide range of reactive aldehydes to their corresponding carboxylic acids, aldehyde dehydrogenases protect cells against stress induced damage [37,64].

#### Amino acids and other metabolism

Our present study revealed that diverse proteins related to nitrogen and amino acids metabolism rapidly respond to salt treatment in rice roots. Enzymes related to ammonium assimilation, aspartate pathway and branched amino acids pathway were up-regulated. They are glutamate dehydrogenase (GDH, spot 314), glutamine synthetase (GS, spot 200), aspartate-semialdehyde dehydrogenase (ASDH, spot 27), aspartate aminotransferase (AAT, spot 67 and 314) and isovaleryl-CoA dehydrogenase (IVDH, spot 68). This is the first report that ASDH and IVDH proteins were up-regulated by salts in rice roots.

Previous reports indicated that GDH and GS are salt stress responsive proteins in rice and Arabidopis roots [5,8,66]. GS catalyze the combination of ammonia and glutamate into glutamine [67]. GDH work as a link between carbon and nitrogen metabolism or deaminate glutamate into ammonium and 2-oxoglutarate. Because glutamate is a precursor of proline, activation of these two enzymes may contribute proline synthesis under salt stress condition [67,68]. Proline is well-known osmo-protectant involved in stress resistant mechanisms in plants. Proline is also known to act as a free radical scavenger [69]. It is noted that two aspartate pathway enzymes, ASDH (spot 27) and AAT (Spot 67 and 314), are increased by salt stress in our data. The aspartate pathway is responsible for the biosynthesis of lysine, threonine, isoleucine, and methionine in most plants and microorganisms. ASDH produce the branch point intermediate between the lysine and threonine/methionine pathways [70]. AAT is an important enzyme involved in carbon and nitrogen metabolism [71]. Li et al. (2010) reported that AAT was induced in rice shoots by combined treatment of ABA and salt [43]. Function of these two aspartate pathway enzymes in plant salt stress is unknown. IVDH (spot 68) was increased by salt treatment. IVDH involved in the catabolism of branched chain amino acids such as leucine and valine [72,73]. Recent report showed that Arabidopsis IVDH mutant increased in 12 of 20 free proteogenic amino acids in seeds, indicating that catabolism plays an important role in regulating levels of branched chain amino acids. In tomato roots, IVDH was reported as aluminum induced proteome [74].

Methylmalonate semi-aldehyde dehydrogenase (MMSDH, spot 259) was down-regulated by salt. MMSDH is a CoA-dependent aldehyde dehydrogenase. It is involved in the distal part of the valine and pyrimidine catabolic pathway and regulation of the long chain fatty acylation in animals and microorganisms. In plant, there is a report that MMSDH is a GA responsive protein and is involved in GA signaling. The rice MMSDH gene and protein was expressed in roots and may play important role in GA induced root cell growth and development [40]. The rapid down-regulation of MMSDH level by salt (Table 1) might be closely correlated with root growth arrest by salt stress. Inorganic phosphatase (spot 82 and 227) was identified as a down-regulated protein by salt. Inorganic phosphatase hydrolyzes PPi and involved in oxidative phosphorylation. Previous reports indicated that it is induced in roots under phosphate starvation condition or GA treatment [75]. It was also identified as a salt-responsive protein in Arabidopsis roots, and as a phosphoprotein in AtSnRK2.8 transgenic plant [5,76]. Over-expression of H^+^PPase has been known to enhance salt tolerance, drought tolerance and phosphorous nutrition in several plants [77,78].

Enoyl-ACP reductase (ENR, spot 258), a fatty acid synthetase, is up-regulated by salt. High expression of ENR is associated with increasing oil concentration of maize [79]. Deficiency in Arabidopsis ENR (MOD1) caused premature cell death in multiple organs [80]. Bacterial ENR homologous transcripts were known to be induced by phosphate stress [81]. However, their function in plant stress response is mostly unknown.

#### Signaling molecules and translation related proteins

A calcium binding protein of calreticulin family (spot 225) was identified as a down-regulated spot by salt (Table 1). Calreticulin (CRT) is multifunctional Ca^2+-^binding protein mainly resident in the endoplasmic reticulum (ER) where it serves as a calcium modulator and chaperone to newly synthesized glycoproteins. Plant CRTs have function in plant growth and development as well as biotic and abiotic stress responses [82]. Previous proteomic reports indicated that CRT protein was up-regulated by salt stress in potato leaves [83]. Jiang et al. (2007) reported that two CRTs were rapidly down-regulated by salt stress in Arabidopsis roots [5]. In rice, CRT protein was induced by GA treatment, but decreased by wound in leaf sheath [84,85]. It is also known that CRT is phosphorylated by CK2 or CDPK [86,87] and dephosphorylated by ABA treatment [88]. There is a report that a wheat CRT was involved in drought stress response [89]. However, function of CRT proteins in plant stress response is mostly unknown.

Salt treatment markedly increased the abundance of two translation elongation factors (Tu and eEF-1 beta, spot 5 and 12) and a translation initiation factor 5A (Spot 383). Ndimba et al. (2005) showed that salt stress affected protein de novo synthesis and several translation initiation factors are up-regulated by hyperosmotic stress [37]. Regulation of the translational machinery is considered to be an important component of cellular stress response [90]. Recently it appeared that Arabidopsis eIF5A3 was involved in supporting growth and to play a regulatory role in the response of plants to sub-lethal osmotic and nutrient stress [91].

### Comparative proteome analysis of wild type and OSRK1 transgenic rice roots

As described, the salt-responsive proteome changes of OSRK1 transgenic rice roots were very tiny compared to that of wild type plants (Table 1). We therefore examined basal expression level of protein spots at unstressed conditions. Interestingly, most of the protein spots identified as early salt-responsive proteins from wild type rice roots were up or down-regulated in transgenic rice to the level of stressed conditions of WT rice even at unstressed condition (see protein ratio in Table 1). Among 52 salt-responsive proteins identified in wild type roots, 38 spots were up-regulated and 10 spots were down-regulated in transgenic rice roots at unstressed condition compared to wild type. Therefore, the protein levels of those spots in unstressed transgenic rice roots were similar to those in salt-treated wild type roots, and salt treatment could not make further differences in transgenic plants. This strongly suggests that salt stress responsive pathway is constitutively turned on by over-expression of OSRK1 in rice roots. In addition, the broad spectrum of functional categories of proteins affected by over-expression of OSRK1 indicates that OSRK1 may act as an upstream regulator of the salt stress response. These include enzymes involved in glycolysis, detoxification and branched amino acid catabolism, GA-responsive proteins, signaling and protein turn over.

In order to know if these protein changes are correlated with gene expression profiles, a comparative microarray analysis were conducted in wild type and transgenic rice roots at 0 h, 3 h or 7 h of salt treatment. NSF rice 45 K oligonucleotide chip was used in this experiment. The microarray analysis revealed 148 up-regulated and 75 down-regulated genes in the transgenic rice roots compared to wild type roots (data not shown). Transcript level of a few genes such as thioredoxin type H (Os07g018600) or DnaK-type molecular chaperone precursor (Os03g0113700) were significantly higher in OSRK1 transgenic rice roots. However, unexpectedly, expression patterns of most identified genes were not positively correlated with our proteome data (Additional file 1: Table S1). This implicates that many changes in proteomes of OSRK1 transgenic rice roots may be attributed to posttranscriptional regulations.

Recently, plant phosphoproteomes were extensively identified in different tissues and in response to various signals. Large-scale comparative phosphoproteomics identified thousands of phosphoproteins from rice and Arabidopsis [92]. It should be noted that many early salt-responsive proteins identified in our proteome analysis were previously known as phosphoproteins in rice. Fructose-bisphosphate aldolase (spot 61), proteasome subunit beta type 2 (spot 112), glutathione S-transferase (spot 112), triose phosphate isomerase (spot 116), ascorbate peroxidase (spot 116, 160, 310 and 351), salt-induced protein (spot 134, 214, and 227), glutamine synthetase (spot 200), methylmalonate-semialdehyde dehydrogenase (spot 259), dnaK-type molecular chaperone (spot 276 and 278) and glyceralde-3-phosphate dehydrogenase (315 and 318) were identical to previous reported phosphoproteoms from rice roots and shoots [36,92-94]. Glyoxalase I (spot 206) and glyceraldehyde 3-phosphate dehydrogenase (spot 315 and 318) were identified as GA-regulated phosphoproteins in rice leaf sheath [94]. Glutamine synthetase (spot 200), fructose bisphosphate aldolase (spot 61) and triosephosphate isomerase (spot 116) were ABA responsive phosphoptorteins in rice leaves [93]. Calreticulin (spot 225) was identified as a phosphoprotein in rice and tobacco cells and phosphorylated by CK2 or CDPK [86,87,95]. In addition to these rice phosphoptroteins, Shin et al. (2007) have identified phosphoproteins from transgenic Arabidopsis over-expressing SnRK2.8 [76]. Among these potential targets of SnRK2.8, ten proteins shared similarity to protein spots up or down-regulated in the OSRK1 transgenic rice roots at unstressed condition (Table 2). They were elongation factor Tu (spot 5), phosphoglycerate kinase (spot 67 and 385), enolase (spot 79), triose phosphate isomerase (spot 116), glutamine synthetase (spot 200), glyoxlase I (glyI, spot 206), glyoxalase II (spot 202 and 265), phosphoglyceromutase (spot 275, 277, 292 and 293), heat shock protein 70 (spot 276 and 278) and glyceraldehyde 3-phosphate dehydrogenase (spot 315 and 318). Taken together, these previous reports suggest that many proteins magnified in OSRK1 transgenic rice roots at unstressed condition are likely to be phosphoproteins.

### Potential SnRK2 phosphorylation sites in the early salt-responsive proteomes

In order to know if these proteins were potential targets of OSRK1, presence of potential phosphorylation sites for SnRK2 kinase were investigated for 38 proteins 1.5 fold up or down regulated in the OSRK1 transgenic rice roots (Table 2). It has been known that plant SnRK2 kinase recognize RXXS/T motif found in ABF/AREB/ABI5 bZIP transcription factors [34,96]. Vlad et al. (2008) suggested LXRXX(S/T) as a recognition motif for Arabidopsis SnRK2.10 using semi-degenerate peptide array [97]. They further found strong phosphorylation motif preference of arginine at -3 position and leucine/isoleucine at -5 position. Table 2 showed that 30 proteins were found to have single or multiple RXX(S/T) motives. The five protein spots known as GA-responsive, i.e., fructose-bisphosphate aldolase (spot 61), inorganic pyrophosphatase (spot 82 and 227), 15 kD organ-specific salt-induced protein (spot 134, 214 and 227), calreticulin (spot 225) and methylmalonate-semialdehyde dehydrogenase (spot 259) contain potential SnRK2 phosphorylation sites. Among 30 proteins, 8 proteins contain (L/I)/XRXXS/T motif, which were isovaleryl-CoA dehydrogenase (spot 68), peroxidase (spot 200), glyoxalase I (spot 206), enoyl-ACP- reductase (spot 258), methylmalonate-semialdehyde dehydrogenase (spot 259), dnaK-type molecular chaperone (spot 276 and 278) and glutamate dehydrogenase (spot 314). It is noted that some of these proteins were found as multiple spots, supporting their post-translational modifications. They were inorganic pyrophosphatase (spot 82 and 227), 15 kDa organ-specific salt-induced protein (spot 134, 214 and 227), 2,3-bisphosphoglycerate-independent phosphoglycerate mutase (spot 275 and 277), dnaK-type molecular chaperone (spot 276 and 278), phosphoglyceromutase (spot 292 and 293) and glyceralde-3-phosphate dehydrogenase (spot 315 and 318). In addition, the potential SnRK2 recognition sites for fructose bisphosphate aldolase (spot 61), proteasome subunit beta type 2 (spot 112) and glyceraldehyde 3-phosphate dehydrogenase (spot 315 and 318) were identical to the phosphopeptides identified from large scale phosphoproteome study [92]. They are potential targets of OSRK1 that need to be further investigated.

**Figure 4:**
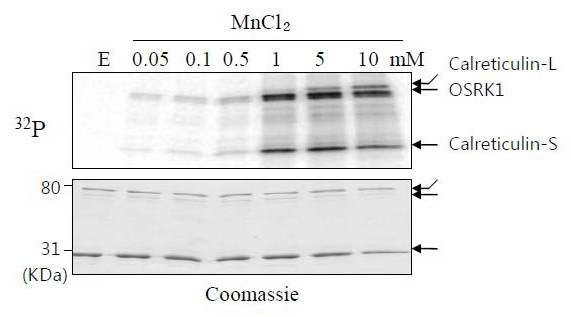
**OSRK1 phosphorylates a rice calreticulin protein**. *In vitro *phosphorylation of GST-OsCRT1 by GST-OSRK1 kinase was assayed in the presence MnCl_2 _at indicated concentrations.

Among these potential targets of OSRK1, phosphorylation of a calcium binding protein, calreticulin (spot 225) was further investigated, because the calreticulin gene (Os07g0246200) was isolated as an OSRK1-interacting clone from yeast two hybrid screening. After affinity purification of GST-fused recombinant protein in *E. coli*, two major bands were appeared (Figure 4) and the 31 kDa lower band (Calreticulin-S) was likely to be a degradation form of calreticulin. The potential SnRK2 phosphorylation site of calreticulin is located at the proximal N-terminus end. Indeed, our *in vitro *kinase assay data showed that OSRK1 could phosphorylate both bands, indicating that calreticulin is a good substrate for OSRK1 kinase (Figure 4).

Our current data implicate that OSRK1 is possibly involved in the diverse metabolic regulation and signaling pathway under salt stress condition by phosphorylation of multiple target proteins. As OSRK1 is involved in ABA signalling pathway, it is likely that these OSRK1-induced proteome/phosphoproteome changes is not only specific to salt stress response, but also involved in multiple stress responses as well. Diverse function of SnRK2 kinase in regulation of glycolysis, detoxification, GA signaling and cell elongation, protein turnover and amino acid catabolism under stress conditions remains to be elucidated.

## Conclusions

Our current proteome data indicated that rice roots rapidly changed broad spectrum of energy metabolism upon challenging salt stress. Anaerobic metabolism is a key component of early salt response of rice roots and glycolysis may play central role. It is noted that several GA-responsive proteomes were clearly down-regulated in rice roots by salt stress. GA is a key regulator of root cell elongation and development of rice. Suppression of GA signaling by salt stress may responsible to the arrest of root growth and developmental under salt condition.

Plant SnRK2 kinase family is a core component of ABA signal transduction pathway and hyperosmotic stress responses. We pursued SnRK2 kinase function in salt response of rice roots by comparative proteomic analysis of OSRK1 transgenic rice. Our proteome data indicated that salt stress responsive diverse metabolic pathways were constitutively activated by over-expression of OSRK1 in rice roots. Most of these proteome changes were not correlated with transcriptional changes. Post-translational regulation, especially phosphorylation is expected to be involved. Many proteins differentially expressed in OSRK1 transgenic rice shared homology to the previously identified phosphoproteins, and contain consensus SnRK2 phosphorylation sites, (L/I)XRXXS/T. One of these potential targets, calreticulin was found to be a good substrate for OSRK1. These results provide new insight for further investigation of SnRK2 function in regulation of metabolism of rice roots under stress condition.

## Methods

### Rice transformation and growth

Rice (*Oryza sativa *cv Nagdong) callus were transformed with *Agrobacterium fumefaciens *LBA4404 carrying the pCAM35S-OSRK1 vector [34]. Transgenic callus were selected and shoots were regenerated in the presence of 30 μg•mL^-1 ^hygromycin. The regenerated plants were transferred to soil and grown in a green house. Homozygous T2 lines were selected based on the hygromycin resistance. Insertion and expression of the transgene was verified by genomic PCR and RT-PCR analysis. For in gel kinase assay, proteome analysis and microarray analysis, 14-day-old seedlings of wild type and OSRK1 transgenic rice grown on 1/2 MS agar medium were transferred to a solution containing 150 mM NaCl. Roots were harvested, frozen in liquid nitrogen, and stored at -80°C until use. For primary root growth analysis, germinated seeds with equally growing primary roots were selected and arrayed on a sheet of pre-wet caligraphy paper. Then the sheets were rolled up and put in a 100 ml beaker containing water or NaCl solution and incubated at 28°C for five days.

### Genomic PCR and RT-PCR analysis

Genomic DNAs and total RNAs were isolated from roots of 14-day-old wild type and transgenic rice seedlings with plant genomic DNA kit (Inclone biotech) or plant RNA kit (Qiagen). For confirmation of transgene insertion, genomic PCR was performed with primers specific to OSRK1 cDNA or hygromycin phosphotransferase (*hpt*) gene. For RT-PCR analysis of transgene expression, first cDNA was synthesized from 5 μg total RNA with oligo (dT) primer using Superscript II reverse transcriptase (Invitrogen). PCR was run for 30 cycles with primers specific to OSRK1 cDNA. As an internal control, transcript level of a rice actin gene (OSJNBa0078A17.12) was monitored. The PCR primers used in these experiments were OSRK1F (5'-atggagaagtacgagctgctc-3'), OSRK1R (5'-tcagctcttctgcaagtcac-3'), HPT5 (5'-agcctgacctattgcatctcc-3'), HPT3 (5'-tgtccgtcaggacattgttgg-3'), ACTIN5 (5'-atcaccattggtgctgag-3') and ACTIN3 (5'-tcctgtgcacaatggatgg-3').

### In gel kinase assay

In-gel kinase assays were performed according to the modified protocol of Ichimura et al. [98]. Proteins were extracted from wild type and transgenic rice roots with extraction buffer (2 mM EDTA, 2 mM EGTA, 2 mM DTT, 25 mM NaF, 0.1 mM Na_3_VO_4_, 50 mM β-glycerophosphate, 1 mM phenylmethylsulfonyl fluoride, 1× protease inhibitor cocktail (Sigma-Aldrich), and 20 mM Tris-HCl, pH 7.5). Proteins (40 μg per lane) were separated on a 10% SDS-PAGE gel containing 0.25 mgmL^-1 ^histone (Sigma H4524, Type III-SS) as a substrate. The gel was washed three times for 30 min with washing buffer (0.5 mM DTT, 5 mM NaF, 0.1 mM Na_3_VO_4_, 0.5 mgmL^-1 ^BSA, 0.1% Triton X-100, and 25 mM Tris-HCl, pH 7.5). For protein renaturation, the gel was washed twice for 30 min with renaturation buffer (1 mM DTT, 5 mM NaF, 0.1 mM Na_3_VO_4_, and 25 mM Tris-HCl, pH 7.5) and further incubated for 16 h at 4°C. The gel was incubated in a reaction buffer (0.1 mM EGTA, 10 mM MgCl_2_, 10 mM MnCl_2_, 1 mM DTT, 0.1 mM Na_3_VO_4_, and 25 mM Tris-HCl, pH 7.5, 50 μCi of [γ-^32^P]ATP and 20 μM cold ATP) for 90 min at room temperature. The gel was washed with 5% TCA and 1% sodium pyrophosphate more than five times for 30 min each, incubated with 10% glycerol, dried and analyzed with a phosphoimage analyzer (Personal Molecular Imager FX system, Bio-Rad, USA).

### Protein extraction and 2-DE analysis

The protein extraction procedure was based on those of Kamo et al. [99] with some modifications. Roots (1.5 g) were ground in liquid nitrogen and precipitated with 10% TCA in acetone with 0.07% mercaptoethanol at -20°C for 1 h, followed by centrifugation for 15 min at 10,000 *g*. The protein pellet was washed with ice-cold acetone containing 0.07% mercaptoethanol at least three times in order to remove contaminants, and lyophilized before 2-DE analysis.

For isoelectric focusing in the first dimension, dried protein samples (1.0 m*g*) were resolved in rehydration buffer (8 M urea, 2.0% CHAPS, 60 mM DTT, 0.5% IPG buffer) and loaded on immobilized linear gradient strips (pH 4-7, 18 cm). Focusing was performed using the following three steps: 500 V for 1 hr, 1000 V for 1 h, and 8000 V for 8 h. The gel strips were equilibrated for 20 min in equilibration buffer (50 mM Tris-HCl, pH 6.8, 6 M Urea, 30% glycerol, 2% SDS, 1% DTT, and 0.002% (w/v) bromophenol blue. The second dimension was run on a 12% polyacrylamide SDS gel using an Ethan Dalt electrophoresis kit (Amersham Biosciences, Sweden). Gels were stained with Coomassie brilliant blue (CBB).

### Image analysis

CBB-stained gels were scanned using a PowerLook III image scanner (UMAX data system). Image treatment, spot detection, and protein quantification were carried out using Progenesis PG240 version 2006 software (Nonlinear dynamics, UK). Spot volumes were determined from at least three gels on which proteins were extracted in triplicate.

### Proteolytic digestion

The stained protein spots excised from the gel were detained with 25 mM ammonium bicarbonate and 50% acetonitrile prior to digestion, and digested with trypsin (Promega, Madison, WI, USA). Gel pieces were swollen in digestion buffer containing 40 mM ammonium bicarbonate and 2 μg trypsin, and incubated at 37°C for 16 h. The peptides were recovered by stepwise extraction with 50 mM ammonium bicarbonate in 50% acetonitrile and 100% acetonitrile. The resulting peptide extracts were pooled and lyophilized in a vacuum centrifuge and stored at -20°C.

### Protein identification by nano-LC-ESI-MS/MS and data analysis

All MS/MS experiments for peptide identification were performed using a nano LC/MS system consisting of an HPLC system (Thermo Scientific, CA, USA) and ESI-quadrupole ion trap MS (LCQ Deca XP-Plus, Thermo Scientific) equipped with a nano-ESI source. Ten μL of sample was loaded by the autosampler onto a C18 trap column (I.D. μm, length 5 mm, particle size 5 μm; LC Packings, Amsterdam, Netherlands) for desalting and concentration at a flow rate of 20 μL•min^-1^. The trapped peptides were then back-flushed and separated on a homemade microcapillary column (150 mm in length) packed with C18 resin (particle size 5 μm) in 75 μm silica tubing (8 μm id orifice). The mobile phases, A and B, were composed of 0% and 90% acetonitrile, respectively, each containing 0.02% formic acid and 0.5% acetic acid. The gradient began at 5% of mobile phase B for 15 min and was ramped to 20% for 3 min, 50% for 32 min, 60% for 5 min, 100% for 5 min and finally held at 100% B for 8 min. The column was equilibrated with 5% mobile phase B for 10 min before the next run. MS and MS/MS spectra were obtained at a heated capillary temperature of 220°C, an ESI voltage of 2.5 kV, and a collision energy setting of 35%. Data-dependent peak selection of the three most abundant MS ions from MS was used. Dynamic exclusion was enabled with a repeat count of 2, a repeat duration of 0.5 min, and 3 min exclusion duration. Mass spectrometer scan functions and HPLC solvent gradients were controlled by the Xcalibur data system (Thermo Scientific). MS/MS mass peak lists were analyzed for *b *and *y *ions using SEQUEST (version 3.3.1, Thermo Scientific) software. SEQUEST was used to match MS/MS spectra to peptides in rice database from the National Center for Biotechnology Information (NCBI: the entry number was 405933) in March 2010. Searches for peptide were first performed with following parameters: a mass tolerance of 2.0 Da on the parent ion and 1.0 Da on the MS/MS, one missed cleavage per peptide was allowed, and modifications of proteins were not taken into account. The validity of peptide/spectrum matches was hence assessed using the SEQUEST defined parameters, cross-correlation score (X_Corr_), and normalized difference in cross-correlation scores (ΔC_n_). Matched peptide had to pass the following filters for provisional identification: 1) ΔC_n _was at least 0.1 and 2) minimum X_Corr _of 1.9, 2.2, and 3.75 for charge states +1, +2, and +3, respectively. SEQUEST automatically saves search results. An SRF file including merging of proteins, filter and sort settings, ratios and protein area/height values was used to select and sort peptide/spectrum matches passing this set of criteria. Proteins were considered detected if they were identified by more than two peptides per spot.

### Purification of GST-fusion protein in E. coli

The ORF of calreticulin gene (Os07g0246200) was PCR-amplified from pAD57, a yeast two hybrid interacting clone of OSRK1, and cloned in frame into pGEX 4T-1 (Amersham). *E. coli *cells (BL21 p*lysisS*, Novagen) carrying the pGEX-fused gene construct were cultured at 37°C until the A_600 _reached 0.5. GST fusion proteins were induced by adding 0.1 mM isopropyl thio-β-D-galactoside (IPTG) and cultures were incubated for 4 h at 37°C. *E. coli *cells were harvested by centrifugation, resuspended in ice-cold TBS (Tris, NaCl) buffer, and lysed by freeze-thaw method. The cell lysates were centrifuged at 20,000 g for 30 min at 4°C and the supernatant was applied to glutathione-Agarose (Peptron, Korea) column. After washing the column with TBS buffer, GST-fusion proteins were eluted with 5 mM glutathione in TBS buffer and used for the kinase assay.

### In vitro kinase assay

*In vitro *kinase assay with OSRK1 protein was performed according to Chae et al. (2007). Briefly, aliquots of GST-fused OSRK1 kinase and calreticulin were incubated in a kinase assay buffer (10 μCi γ-^32^P-ATP, 20 mM Tris-HCl pH 7.0, MnCl_2_) for 30 min at 30°C. The reaction was stopped by adding 5X SDS sample buffer, boiled immediately for 5 min, and products were analyzed by SDS-PAGE. Gels were stained with Coomasie Brilliant Blue R-250, dried, and analyzed with a phosphoimage analyzer (Personal Molecular Imager FX system, Bio-Rad, USA).

### Microarray analysis

Total RNAs from rice roots were isolated with plant RNA kit (Qiagen). RNA length distribution and integrity were assessed by capillary electrophoresis with fluorescence detection (Agilent Bioanalyzer 2100) using the Agilent Total RNA Nano chip assay for presence of 28S and 18S rRNA bands. Fluorescent-labeled cRNA for Oligo micorarray analysis was prepared by amplification of total RNA in the presence of aminoallyl-UTP followed by the coupling of Cy3 or Cy5 dye (AmershamPharmacia, Uppsala, Sweden). NSF 45 K Oligo Microarray kit was hybridized with the fluorescently labeled cRNA at 42°C for 16 h and then washed. DNA chips were scanned using GenePix 4000B (Axon Instruments, Union City, CA). Scanned images were analyzed with GenePix Pro 3.0 software (Axon Instruments, Union City, CA) to obtain gene expression ratios. Transformed data were normalized using the Lowess procedure.

## Competing interests

The authors declare that they have no competing interests.

## Authors' contributions

MHN performed proteomic analysis including its design, coordination, analysis of the data, and drafted the manuscript. KMK performed 2-DE and gel image analysis. JBS and KC conceived the LC-MS analysis and analysed the proteome data. DYK and BGK performed rice transformation. SMH and WJP performed transgenic rice analysis and in gel kinase assay. ISY conceived of overall experimental design and manuscript preparation. All authors read and approved the final manuscript.

## Supplementary Material

Additional file 1 **Table S1**. Fold changes of transcript level of genes correspond to protein spots showing more than 1.5 fold change in OSRK1 transgenic rice roots at unstressed condition. Click here for file
