# Supplementary material for: Comparative proteomic analysis of early salt stress-responsive proteins in roots of SnRK2 transgenic rice
Source: Proteome Sci. 2012 Mar 31;10:25. doi: 10.1186/1477-5956-10-25 (PMC3364906; doi:10.1186/1477-5956-10-25)
Supplement: Additional file 1 — Table S1. Fold changes of transcript level of genes correspond to protein spots showing more than 1.5 fold change in OSRK1 transgenic rice roots at unstressed condition. [file 1477-5956-10-25-S1.DOC]

# Additional files

### Additional file 1

**Table S1. Fold changes of transcript level of genes correspond to protein spots showing more than 1.5 fold change in OSRK1 transgenic rice roots at unstressed condition.**

| Spot^a^ | Protein Identification | NCBI  Accession # | RAP ID | 0 h | FC^b^  3 h | 7 h | Protein ratio^d^  3h |
| --- | --- | --- | --- | --- | --- | --- | --- |
| 5 | Elongation factor Tu | gi|21685576 | Os03g0851100 | 1.7 | 1.3 | 0.8 | I^e^ |
| 27 | Aspartate-semialdehyde dehydrogenase | gi|113549818 | Os03g0760700 | 0.9 | 1.1 | 0.9 | 5.46 |
| 61 | Fructose-bisphosphate aldolase | gi|790970 | Os01g0905800 | 1.5 | 1.7 | 1.0 | 0.82 |
| 67 | Aspartate aminotransferase | gi|29468084 | Os02g0797500 | NA^c^ | NA^c^ | NA^c^ | 3.64 |
| 68 | Putative isovaleryl-CoA dehydrogenase | gi|113578072 | Os05g0125500 | 1.4 | 1.3 | 0.9 | 2.53 |
| 69 | Putative acetyl-CoA C-acetyltransferase | gi|113630918 | Os09g0252100 | 1.4 | 1.6 | 0.8 | 6.40 |
| 76 | Dihydrolipoamide dehydrogenase precursor | gi|113532449 | Os01g0328700 | 1.3 | 0.9 | 0.9 | I^e^ |
| 77 | Transketolase | gi|227468492 | Os06g0133800 | 1.7 | 1.4 | 0.9 | 18.68 |
| 79 | Similar to enolase | gi|115478881 | Os09g0375000 | 1.6 | 1.7 | 1.1 | 3.14 |
| 82, 227 | Putative inorganic pyrophosphatase | gi|113537770 | Os02g0768600 | 0.9 | 1.1 | 1.2 | 0.42, 0.44 |
| 112 | Glutathione S-transferase | gi|31433227 | Os10g0530900 | 0.9 | 1.0 | 0.9 | 0.28 |
| 112 | Proteasome subunit beta type 2 | gi|17380213 | Os03g0695600 | 1.4 | 1.7 | 1.1 | 0.28 |
| 112 | Putative chaperonin21 precursor | gi|51090748 | Os06g0196900 | 1.3 | 1.5 | 0.9 | 0.28 |
| 116 | Triose phosphate isomerise | gi|553107 | Os01g0147900 | 1.3 | 1.2 | 0.9 | 2.40 |
| 124 | Formate dehydrogenase, mitochondrial precursor | gi|21263611 | Os06g0486800 | 1.6 | 1.3 | 0.7 | 4.13 |
| 125 | Glutathione S-transferase II | gi|3746581 | Os01g0764000 | 1.9 | 2.3 | 1.5 | 2.63 |
| 134 | Oryzacystatin | gi|1280613 | Os01g0803200 | 1.0 | 2.8 | 1.2 | 0.22 |
| 134, 214, 227 | 15 kda organ-specific salt-induced protein | gi|256638 | Os01g0348900 | 1.2 | 2.5 | 2.0 | 0.22,0.48, 0.44 |
| 160 | Ascorbate peroxidise | gi|1321661 | Os03g0285700 | 1.6 | 2.3 | 1.5 | 0.41 |
| 200 | Peroxidase | gi|257657027 | Os05g0162000 | 1.2 | 1.7 | 1.5 | 1.72 |
| 200 | Glutamine synthetase | gi|124052115 | Os02g0735200 | 1.5 | 1.5 | 1.1 | 1.72 |
| 202, 265 | Putative glyoxalase II | gi|113533338 | Os01g0667200 | 0.7 | 0.9 | 0.9 | 1.47, 1.90 |
| 206 | Glyoxalase I | gi|113623141 | Os08g0191700 | NA^c^ | NA^c^ | NA^c^ | 4.46 |
| 213 | Thioredoxin Type H | gi|82407383 | Os07g0186000 | 2.0 | 6.9 | 2.0 | 3.20 |
| 225 | Calreticulin | gi|6682833 | Os07g0246200 | 0.9 | 1.0 | 1.1 | 0.26 |
| 258 | Putative enoyl-ACP reductase | gi|113623526 | Os08g0327400 | 1.1 | 1.0 | 1.0 | 2.92 |
| 259 | Methylmalonate semi-aldehyde dehydrogenase | gi|113610618 | Os07g0188800 | NA^c^ | NA^c^ | NA^c^ | 0.27 |
| 275,277 | 2,3-bisphosphoglycerate-independent phosphoglycerate mutase | gi|257353836 | Os05g0482700 | 2.4 | 1.7 | 0.6 | 2.07, 2.33 |
| 276, 278 | DnaK-type molecular chaperone precursor | gi|257307253 | Os03g0113700 | 2.1 | 3.2 | 0.9 | 2.78, 4.47 |
| 292, 293 | Phosphoglyceromutase | gi|257353838 | Os01g0817700 | 2.4 | 1.6 | 0.6 | 2.66, 1.90 |
| 314 | Glutamate dehydrogenase | gi|33242905 | Os03g0794500 | 1.2 | 1.4 | 0.9 | 3.08 |
| 314 | Aspartate aminotransferase | gi|215768565 | Os03g0157900 | 1.2 | 0.8 | 1.0 | 3.08 |
| 315, 318 | Glyceralde-3-phosphate dehydrogenase | gi|968996 | Os08g0126300 | 2.1 | 1.4 | 1.4 | 2.15, 3.06 |
| 385, 67 | Phosphoglycerate kinase | gi|113596357 | Os06g0668200 | 1.4 | 1.7 | 1.0 | I^e^, 3.64 |
| 383 | Translation initiation factor 5A | gi|113611710 | Os07g0597000 | 1.6 | 1.8 | 1.1 | I^e^ |
| 386 | Pathogen-related protein | gi|16589076 | Os03g0300400 | 1.5 | 1.6 | 1.4 | 6.70 |
| 390 | Aldehyde dehydrogenase | gi|8163730 | Os06g0270900 | 2.1 | 1.6 | 0.7 | 10.85 |
| 392 | Hypothetical protein | gi|14192878 | Os07g0609000 | 0.7 | 0.9 | 0.9 | 18.95 |
| Control | OSRK1 | ND^f^ | Os02g0551100 | 6.9 | 7.3 | 6.6 | ND^f^ |

^a^Spot numbers correspond to the spots in figure 2.

^b^FC; Fold change of gene expression level in OSRK1 transgenic rice roots compared to wild type after NaCl treatment for 0 h, 3 h or 7 h, respectively.

^c^NA; Not appeared in the microarray data

^d^Spot-volume ratio of WT to OSRK1, calculated from the mean spot volumes in 3 h control of WT and OSRK1 groups.

^e^I; Spots were not appeared in WT groups, but detected in OSRK1 groups

^f^ND; Not identified from the proteome data
